# A case report of left lower lobe segmentectomy for pulmonary metastasis from retroperitoneal liposarcoma

**DOI:** 10.1016/j.ijscr.2019.07.059

**Published:** 2019-07-26

**Authors:** Kazuhiro Yoshida, Masakazu Yoshida, Minoru Haisa, Takuro Yukawa, Yasumasa Monobe, Yoshio Naomoto, Takuya Fukazawa, Tomoki Yamatsuji

**Affiliations:** a Department of General Surgery, Kawasaki Medical School, Okayama, 700-8505, Japan; b Department of Pathology 1, Kawasaki Medical School, Okayama, 700-8505, Japan

**Keywords:** Retroperitoneal liposarcoma, Pulmonary metastasis, Limited resection

## Abstract

**Background:**

The principle treatment for retroperitoneal liposarcoma is surgical resection, however there are many cases of recurrence. In addition to local recurrence, retroperitoneal liposarcoma, particularly dedifferentiated liposarcoma is known to occasionally cause lung metastases.

**Case report:**

A 72-year-old woman with a diagnosis of retroperitoneal liposarcoma and probable right upper lobe early pulmonary adenocarcinoma underwent sequential local tumor resection and right upper lobectomy. Twenty months after liposarcoma resection, a computed tomography (CT) scan of the chest revealed a nodule located in the left lower lobe. A CT-guided biopsy was performed and she was subsequently diagnosed with pulmonary metastasis from retroperitoneal liposarcoma. The nodule enlarged chronologically, however a left lower lobectomy could not be performed because respiratory function after the right upper lobectomy was not sufficient. Therefore, in order to preserve the left superior segment (S6), the basal segments (S8+S9+S10) were resected. Seven months after the surgery, she is living a self-reliant life without recurrence of liposarcoma.

**Conclusion:**

Here we have reported a case of pulmonary metastasis from retroperitoneal liposarcoma following limited surgery. In cases where respiratory function is limited, lower lobe segmentectomy can be an effective treatment. For the treatment of a single pulmonary metastasis from retroperitoneal liposarcoma, metastasectomy was considered to be effective as long as no local recurrence was seen after initial primary tumor resection.

## Highlights

• Many recurrences occur after surgical resection of retroperitoneal liposarcoma.
• Dedifferentiated liposarcoma in particular occasionally causes lung metastases.
• In the case of no local recurrence, metastasectomy might be effective.

## 1 Background

In this report, we describe a case of pulmonary metastasis from dedifferentiated liposarcoma (DDLS) that has had no local recurrence following surgical resection. Retroperitoneal liposarcomas (RPLPS) are rare neoplasms that account for approximately 30% to 50% of all retroperitoneal sarcomas [1]. There are four types of liposarcoma and the two most common types in the retroperitoneum are well-differentiated liposarcoma (WDLPS) and DDLPS. WDLPS is known as a locally aggressive tumor and is characterized by repeated local recurrences. The local recurrence rate has been reported to be 40% to 60%. DDLS also has a higher local recurrence rate (40% to 80%) than WDLS and six times the risk of death [2]. Complete surgical resection with negative margins is the only curative treatment for these kinds of tumors. Compared to WDLS, DDLPS has more distant metastatic potential and the distant metastasis rates range from 10% to 15% [3]. It has been reported that the lung is the most common site for distant metastasis of DDLPS [4]. The work has been reported in line with the SCARE 2018 statement [5]

## 2 Case report

**Fig. 1:**
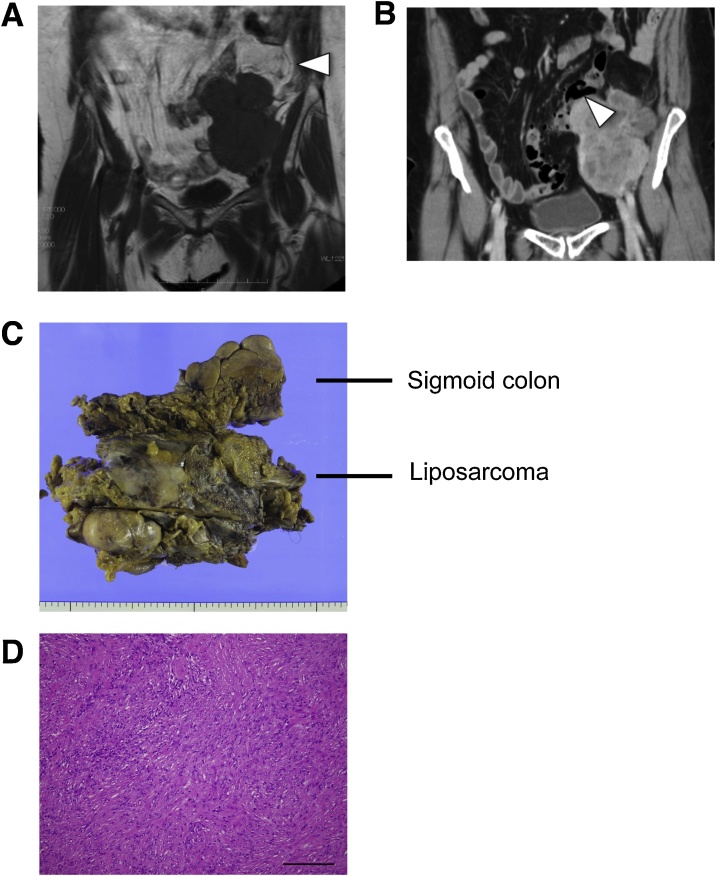
**A**. Magnetic resonance imaging showing a voluminous tumor on the left side of the pelvis and a retroperitoneal liposarcoma was suspected. Arrow head indicates a well-differentiated component. **B**. A computed tomography of the abdomen indicating that the tumor might be invading the descending colon. Arrow head indicates sigmoid colon invasion. **C**. Surgical specimen with in-block resection of DDLS and a sigmoid colon. DDLS appears to be 15 × 10 × 7.5 cm in size. Arrows indicate a resected sigmoid colon. **D**. The histopathological diagnosis was dedifferentiated liposarcoma (DDLS). Scale bar = 200 μm.

**Fig. 2:**
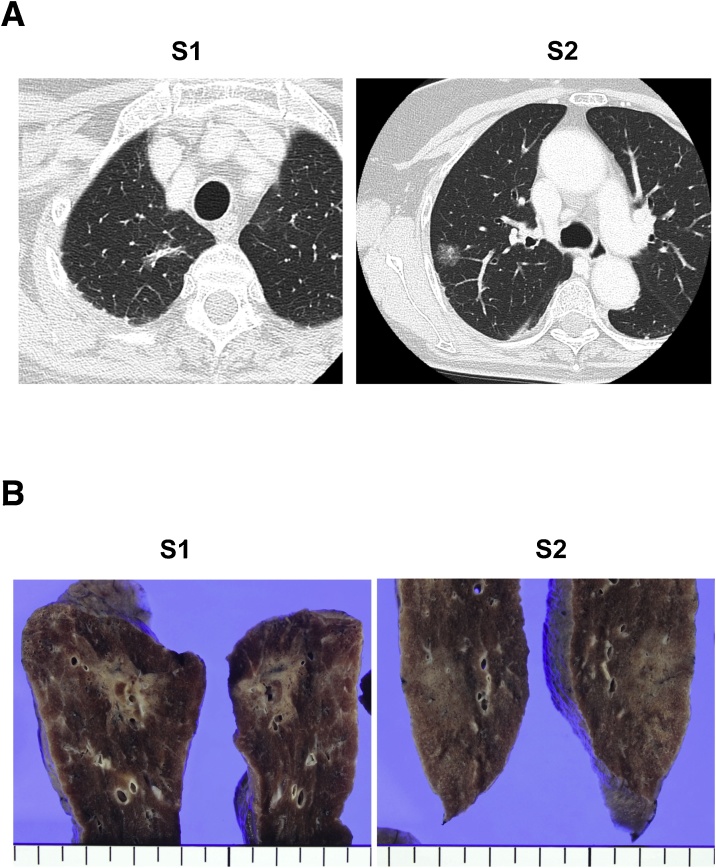
**A**. A computed tomography of the chest showing a band-like shadow with ground-glass opacity (GGO) in the apical segment (S1) of the right upper lobe of the lung. A GGO that contains a solid component was also detected in the posterior segment (S2). Both these two regions were considered as early stage lung cancer (S1: cT1aN0M0 Stage1A1, S2 cT1bN0M0 Stage1A2). **B**. Macroscopic findings of the tumor. Pathological diagnosis of the S1 region was non-invasive adenocarcinoma (pT1bN0M0, Stage1A2). The S2 region was diagnosed as invasive adenocarcinoma (pT1aN0M0, Stage1A1).

A 72-yr old female visited a local hospital complaining of left lower abdominal discomfort and constipation. An abdominal computed tomography (CT) confirmed a retroperitoneal tumor and the patient was referred to our hospital for further examination. Magnetic resonance imaging (MRI) of the abdomen showed a solid mass measuring 13 cm in diameter on the left side of the pelvis and a liposarcoma containing a well-differentiated component was diagnosed (Fig. 1A). A high-resolution computed tomography (HRCT) of the abdomen revealed that the tumor was suspected to invade the left ureter and descending colon (Fig. 1B). Moreover, HRCT of the chest detected two ground-glass opacities (GGO) in the apical segment (S1) and posterior segment (S2) of the right upper lobe of the lung and was considered to be early stage lung cancer (Fig. 2A). Treatment was carried out by retroperitoneal liposarcoma and retroperitoneal tumor resection after ureteral stent placement by urologists. Intraoperative findings showed that the tumor displaced the descending colon which was therefore partially resected. The tumor size was 13 × 8 x 7.5 cm and postoperative pathological findings indicated DDLPS with invasion to the sigmoid colon (Fig. 1C, D). Four months after the retroperitoneal liposarcoma resection, a right upper lobectomy for suspected lung cancer was performed via video-assisted thoracoscopic surgery (VATS) (Fig. 2B).

**Fig. 3:**
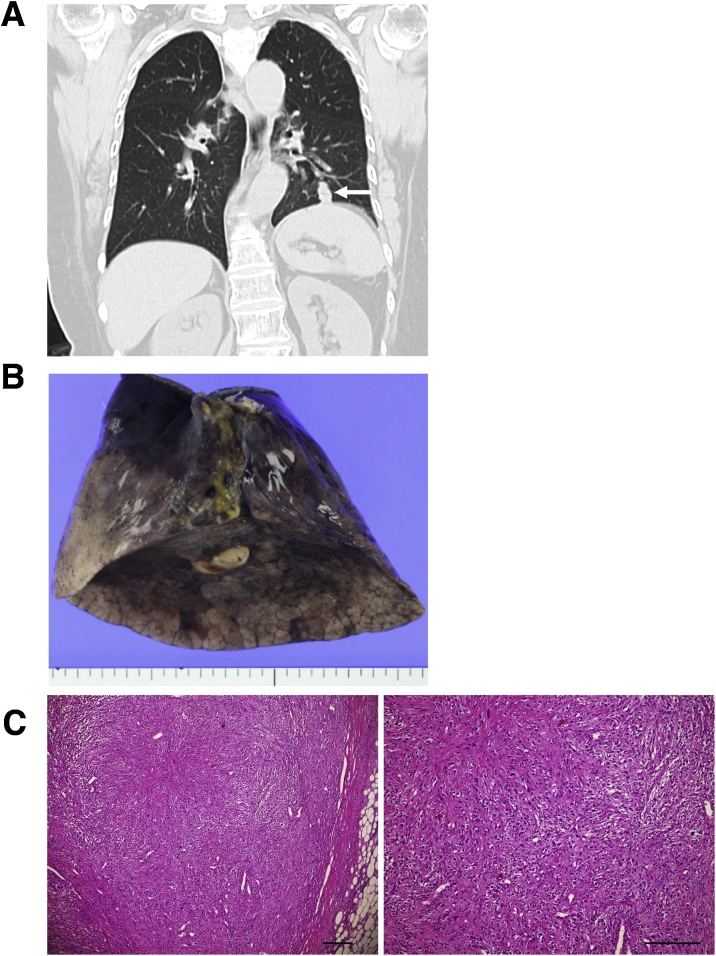
**A**. A HRCT of the chest detected a nodule with a maximum diameter of 22 mm in the left lower lobe of the lung. **B**. Macroscopic findings of the metastatic tumor. The nodule appears white and lobular. Pathologically, no pleural invasion was observed. **C**. Pathological diagnosis of the tumor was metastasis from DDLS. Scale bar = 200 μm.

After lung cancer surgery, the patient was followed-up as an outpatient. Twenty months after the initial surgery, a HRCT of the chest detected a tumor with maximum diameter of 22 mm in the left lower lobe of the lung (Fig. 3A). Because the tumor was located in the peripheral lung, CT-guided fine needle biopsy was performed rather than transbronchial bronchial lung biopsy (TBLB) [6]. Pathological findings indicated pulmonary metastasis from dedifferentiated liposarcoma and the tumor tended to grow rapidly. The result of a respiratory function test on the patient showed that her current respiratory function was not sufficient to allow a left lower lobectomy, therefore, in order to preserve the superior segment of the lower lobe (S6), a left basal segmentectomy (S8+S9+S10) [7] was conducted (Fig. 3B). Pathological findings showted DDLPS with invasion to the sigmoid colon (Fig. 3C). The postoperative course was uneventful and she was discharged on the 16th day after the operation. There is no sign of recurrence of the tumor seven months after discharge. She is undergoing outpatient rehabilitation, and she is able to live an independent life without home oxygen therapy.

## 3 Discussion

RPLPS is not often detected until it becomes enlarged because it does not demonstrate any characteristic symptoms. RPLPS is sometimes incidentally detected during abdominal examination or when abdominal imaging studies are performed for other purposes. The majority of patients with RPLPS are diagnosed at an advanced disease stage, therefore the tumors often grow to a large size and have invaded adjacent organs upon diagnosis. RPLPS typically occurs in patients of 40–60 years, with a 1:1 ratio between male and female patients [8]. The World Health Organization classification of liposarcoma includes four recognized histologic types: well differentiated (WDLPS), dedifferentiated (DDLPS), myxoid and pleomorphic and the two most common types in the retroperitoneum which are WDLPS and DDLPS [9]. These two varieties share the molecular hallmark of *MDM2* gene amplification which differentiates these from other retroperitoneal tumors [10]. Unique to liposarcoma, these two types are sometimes found within the same tumor juxtaposed to each other [11].

It has been reported that despite an aggressive surgical approach, over 80% of patients with DDLPS will recur locally and 30% will metastasize to distant sites within 3 years of diagnosis with the lung being the most common site of metastasis for DDLPS [12,13]. Billingsley et al. have advocated complete resection of the metastatic site as a positive prognosis factor for pulmonary metastasis from soft tissue sarcoma and a longer disease-free interval (>12 months) [4]. Moreover, the Trans-Atlantic Retroperitoneal Sarcoma Working Group (TARPSWG) has reported that complete resection of lung metastasis without adjuvant therapy is not an absolute contraindication and occasionally has prolonged survival in selected patients [14]. In the present case, no recurrence was observed after retroperitoneal tumor resection, therefore the upper lobectomy for early lung cancer was sequentially performed four months after the initial operation.

Seven months have passed since the last operation, but no recurrence has occurred. However, in order to detect local recurrence and distant metastasis early, outpatient follow-up is important. Presently, surgical intervention with cleanly resected margins is the primary treatment for pulmonary metastasis from liposarcoma. Nevertheless, antitumor effects have been reported using various kinds of anticancer agents or molecular targeting drugs for unresectable cases of liposarcoma [[15], [16], [17]], but no effective regimen has yet been established. In the future, the establishment of a multidisciplinary treatment including radiation therapy, molecular target therapy and immune therapy is expected to treat liposarcoma, particularly for patients with initially unresectable advanced liposarcoma and recurrence after surgery [18,19].

## 4 Conclusions

In conclusion, we report a case of pulmonary metastasis from retroperitoneal dedifferentiated liposarcoma. Left basal segmentectomy was conducted because there was only a single tumor in both lungs and no local recurrence was detected after the initial retroperitoneal liposarcoma resection. Metastasis of retroperitoneal DDLPS outside the abdominal cavity is rare. Further large studies should be conducted to evaluate the effectiveness of pulmonary resection for the treatment of retroperitoneal liposarcoma with lung metastasis.

## Funding

We have no sponsors for this research.

## Ethical approval

A case report is exempt from ethnical approval in our institution.

## Consent

We have obtained a written informed consent from the patient for publication of this case report and accompanying images. In this case report, we tried to omit unnecessary identifying details as much as possible.

## Author contribution

T.F., K.Y. and T Yukawa wrote the manuscript. M.Y., M.H., Y.M., Y. N. and T Yamatsuji provided scientific discussion. All authors contributed to the discussion and review of the manuscript.

## Registration of research studies

N/A.

This is a case report and not any observational study.

## Guarantor

The professor emeritus of our department has responsibility for the work and/or the conduct of the study, had access to the data, and controlled the decision to publish.

## Provenance and peer review

Not commissioned, externally peer-reviewed.

## Declaration of Competing Interest

We have no financial relationships to disclose.
